# Knowledge, Attitude, and Practice toward Skin Cancer among Patients of Dermatology Clinics and Medical Students/General Practitioners

**DOI:** 10.1155/2024/9081896

**Published:** 2024-05-20

**Authors:** Moein Baghani, Reza M. Robati, Nikoo Mozafari, Matin Baghani, Martin Kassir, Fatemeh Sheibani, Vahid Mansouri

**Affiliations:** ^1^Skin Research Center, Shahid Beheshti University of Medical Sciences, Tehran, Iran; ^2^School of Medicine, Shahid Beheshti University of Medical Sciences, Tehran, Iran; ^3^Department of Dermatology, Loghman Hakim Hospital, Shahid Beheshti University of Medical Sciences, Tehran, Iran; ^4^Worldwide Laser Institute, Dallas, Texas, USA; ^5^Laser Application in Medical Sciences Research Center, Shahid Beheshti University of Medical Sciences, Tehran, Iran; ^6^Proteomics Research Center, Faculty of Paramedical Sciences, Shahid Beheshti University of Medical Sciences, Tehran, Iran

## Abstract

This cross-sectional study assessed the knowledge, attitude, and practices (KAP) regarding skin cancer among dermatology clinic patients, medical students, and general practitioners (GPs) in Tehran, Iran. The researchers collected data using a validated questionnaire administered online, measuring KAP on scales of 0–31, 0–16, and 0–28, respectively, with scores above 16, 8, and 14 indicating “good” levels. Of 2243 participants (mean age 28 years), 59.4% had good knowledge, 19.8% had good attitudes, 31.8% had good practices, and 29.8% had good overall KAP. Medical students/GPs scored higher on knowledge and attitudes, while patients scored better on practices. Knowledge, attitudes, and practices were positively correlated in professionals but inversely correlated in patients. The findings suggest that while knowledge was moderate, attitudes and behaviors remained poor, particularly among patients. Immediate interventions are needed to improve attitudes and prevention practices, as public health initiatives must focus on positively influencing both to translate knowledge into meaningful action and find the reasons why good knowledge may not always lead to good practice. These findings underline the need for targeted interventions to bridge the gap between knowledge and preventive behaviors, to effectively reduce the burden of skin cancer in the population.

## 1. Introduction

Skin cancer is a growing public health concern, with three main types: squamous cell carcinoma, basal cell carcinoma, and malignant melanoma. Squamous cell carcinoma and basal cell carcinoma are the most common forms of skin cancer, while malignant melanoma is less frequent but remains the primary cause of mortality from skin cancer. Early detection and treatment of all forms of skin cancer are crucial for improving patient outcomes. Skin cancer occurs more frequently in men and among persons with a light skin tone, persons who use indoor tanning beds, and persons with a history of sunburns or previous skin cancer. Specific risk factors for melanoma include having a dysplastic nevus, multiple (≥100) nevi, and a family history of melanoma. Risk of melanoma also increases with age. The clinical visual skin examination evaluates skin lesions using the “ABCDE rule,” which includes looking for the following features: asymmetry, border irregularity, nonuniform color, diameter >6 mm, and evolving over time. The “ABCDE rule” is a simple way to differentiate malignant melanoma from common melanocytic moles. However, the diagnosis of malignant melanoma should be confirmed with the histopathology assessment of the suspected lesions [1–3].

The incidence of skin cancer continues to rise globally, including in the Middle East region, where it is the most prevalent cancer, and in Iran has a high burden due to the country's year-round high levels of sunshine [3–6].

The health and economic impact of skin cancer is substantial and increasing. Studies have shown that the direct and indirect costs of skin cancer treatment place a significant strain on healthcare systems [7]. Therefore, interventions that promote sun-protective behaviors and early detection of skin cancer could lead to significant savings and better health outcomes [8, 9].

Exposure to ultraviolet radiation (UVR), particularly from the sun, is the primary risk factor for the development of skin malignancies [10]. Effective sun-protective measures, such as the regular use of sunscreen, wearing protective clothing, and seeking shade, have been shown to reduce the risk of squamous cell carcinoma and melanoma [8, 11]. The majority of skin cancer prevention initiatives are predicated on the idea that a better understanding and knowledge of the disease would influence individuals' attitudes and sun-protective behaviors. Research has indicated that knowledge alone does not necessarily translate into the adoption of sun-safe practices [12, 13].

This study aims to evaluate the knowledge, attitudes, and practices (KAP) of dermatology patients and medical students/general practitioners (GPs) toward skin cancer. Understanding the current state of KAP in these groups is crucial for developing targeted educational and intervention strategies to improve skin cancer prevention and early detection efforts.

## 2. Materials and Methods

### 2.1. Participants and Study Design

This cross-sectional study included medical students from Shahid Beheshti University of Medical Sciences, general practitioners (GPs) practicing in the region, and patients attending the Dermatology Clinic of Loghman Hospital, Tehran, Iran. Patients were mainly visiting for aesthetic practices and general dermatologic complaints, such as eczema, urticaria, and psoriasis. Participants were recruited by convenience sampling. Dermatology patients were eligible if they were aged 18 years or older and able to understand and complete the survey. Individuals with language barriers, cognitive impairments, or who declined to provide informed consent were excluded from the study. The ethical committee of the Shahid Beheshti University of Medical Sciences approved the study protocol (Ethical cod IR.SBMU.SRC.REC.1399.009).

The required sample size was calculated based on a statistical power analysis to ensure adequate power to detect differences between groups. With a type I error rate of 0.05 and a desired power of 90%, the minimum required sample size was estimated to be 290 participants in each group (patients and medical students). This sample size calculation was performed prior to starting data collection to determine the number of subjects needed to reliably compare knowledge scores among the participant groups.

### 2.2. Data Collection

A researcher-made questionnaire was administered using Google Forms, an online survey platform. The items were primarily developed based on the Mahmoodabad et al. questionnaire, based on a review of the existing literature and input from a panel of experts in dermatology and public health, which has been established as reliable and validated by a pilot study and confirmed with a Cronbach alpha test (*a* = 0.812) [14].

The survey link was disseminated to the target participant groups through multiple channels. For medical students, the link was shared with the administration of Shahid Beheshti University of Medical Sciences, who then distributed it to students via e-mail and university communication channels. For GPs, the link was shared with the Tehran Medical Association, which forwarded it to GPs practicing in the region via e-mail and professional networks. For dermatology patients, the clinic staff at Loghman Hospital approached eligible patients during their visits and offered them the opportunity to participate using their personal smartphones or clinic computers.

In all cases, potential participants were informed about the purpose of the study, the voluntary nature of participation, and the measures taken to ensure confidentiality of the data. Participants who agreed to take part in the survey were able to access the Google Forms link and complete the questionnaire online. The survey platform was configured to prevent multiple submissions from the same individual.

### 2.3. Measurement of Variables

The 52-item questionnaire consisted of four sections:

1. Demographic and general characteristics (11 items) (Table 1)
2. Knowledge about skin cancer risk factors and prevention (15 items, score range: 0–31) (Supplementary Table 1)
3. Attitudes toward skin cancer (16 items, score range: 0–16) (Supplementary Table 2)
4. Self-reported sun-protective practices (9 items, score range: 0–28) (Supplementary Table 3)

### 2.4. Demographic and General Characteristics Variables

These items included age, sex, education level, group (patients or medical students/general practitioners), and healthcare-related occupation. Additionally, participants in healthcare-related occupations were asked if other people had sought knowledge or information about health-related topics by asking them. History of skin cancer in the subject or his/her family, sources of skin cancer information, and the Fitzpatrick skin type were assessed using a self-reported classification system that categorizes skin types into six types (I–VI) based on an individual's response to sun exposure and natural skin pigmentation. For the dermatology clinic patients, clinic staff were available to provide clarification or assistance if participants had any confusion about correctly identifying their Fitzpatrick skin type. This helped to ensure accurate self-reporting of this important variable, particularly among the patient group who may have had less familiarity with the skin type classification system.

### 2.5. Knowledge Variables

The questions were designed as multiple choices and some questions such as the potential factors increasing the risk of skin cancer, more than one correct answer was possible, and the number of correct answers would determine the total score of that question. Each correct answer awarded one point. Score ranging from 0 to 31. (Score 17–31 was considered good knowledge.)

### 2.6. Attitude Variables

These variables were measured by a Likert scale, three responses (Agree-Disagree-No idea) with certain expressions negatively directed to prevent leading effects. Scores range from 0 to 16. (Scores 9–16 were considered good attitude.)

### 2.7. Performance or Practice Variables

Answers to the questions of this section were either positive or negative as each positive answer would be awarded with one point and some answers would have been awarded up to four points as showed more consistent positive performances. Scores range from 0 to 28. (Scores 15–28 were considered good performance.)

### 2.8. Data Analysis

We used mean and standard deviation to describe continuous variables and frequency and percentage to describe categorical variables. According to the central limit theorem [15], since the sample size was over 30 in each group, we utilized the independent *t*-test to compare continuous variables between patients and medical students/GPs. Moreover, to assess the correlation among knowledge, attitude, and practice of the participants toward skin cancer, we used Spearman's Rho since these data were abnormally distributed. To determine the association of participants' general characteristics with their knowledge, attitude, practice, and overall KAP toward skin cancer, scores >16, >8, >14, and >38 were regarded as good, respectively. Accordingly, logistic regression analysis was used to ascertain the related factors. The factors with *P* values of <0.2 in univariable analyses were included in the multivariable regression model. All data were analyzed using the SPSS software (version 25.0). *P* values <0.05 were regarded as statistically significant.

## 3. Results and Discussion

### 3.1. Results

Of 2264 filled questionnaires, 21 were incomplete; therefore, the information from 2243 participants was included in the final analysis. Their mean age was 28.37 ± 8.49 years. Of these, 1057 (47.1%) were males and 1186 (52.9%) were females. 290 (12.9%) of the responders were medical students/GPs. Table 1 demonstrates the general characteristics of the study subjects. Seventy subjects (3.1%) had a history of skin cancer, while a family history of skin cancer was present in 83 (3.7%). Type III (30%) was the most common Fitzpatrick skin type.

Social media (e.g., Instagram) (73.4%) was the source of information about skin cancer in the majority of participants. Response patterns varied between patient and medical respondent groups for certain sources like family/friends and books/magazines, likely due to overlap between categories (Table 1).

**Figure 1:**
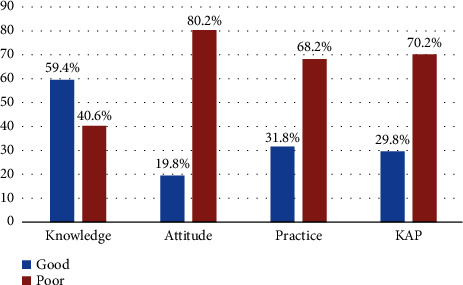
Participants' knowledge, attitude, practice, and overall KAP.

**Figure 2:**
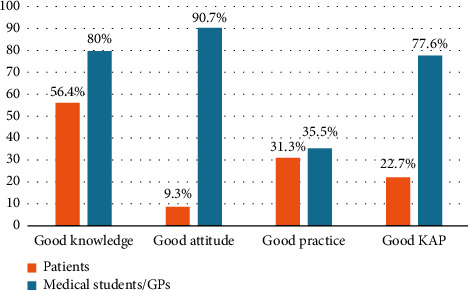
Comparison of knowledge, attitude, practice, and KAP between patients and medical students/GPs.

Good knowledge, attitude, practice, and overall KAP were observed in 59.4%, 19.8%, 31.8%, and 29.8% of the participants (Figure 1), with their comparison between patients and medical students/GPs depicted in Figure 2. The mean scores of knowledge and attitude toward skin cancer were significantly higher in medical students/GPs compared to the patients (*P* < 0.001); however, the mean score of practice toward skin cancer was significantly better in patients (*P* < 0.001) (Table 2, Supplementary Tables 1–3).

Knowledge, attitude, and practice toward skin cancer were positively correlated in medical students/GPs (*P* < 0.001). Nonetheless, among patients, knowledge was negatively correlated with attitude (*r* = −0.142, *P* < 0.001), but positively correlated with practice (*r* = 0.298, *P* < 0.001). Moreover, attitude was inversely correlated with practice in patients (*r* = −0.062, *P* < 0.001) (Table 3).

On the other hand, age, sex, education, having a health-related occupation, and a personal and family history of skin cancer were significantly associated with patients' overall KAP toward skin cancer, while only age, sex, and education were significantly associated with that of medical students/GPs (Table 4).

### 3.2. Discussion

Among the most prevalent malignancies today, skin cancer has rising incidence and fatality rates, and there are several precautionary measures that may be taken to lower the risk of developing skin cancer, with using sunscreen and limiting exposure to sunlight, particularly during peak hours, being the most effective ones [16, 17]. Given the numerous identified risk factors for skin cancer, the World Health Organization has adopted strategies to prevent skin cancer by expanding people's understanding of the disease via health education, changing attitudes toward it, and enhancing performance [18].

Good knowledge, attitude, practice, and overall KAP toward skin cancer were observed in 59.4%, 19.8%, 31.8%, and 29.8% of this study's participants. Knowledge and attitude toward skin cancer were significantly higher in medical students/GPs compared to dermatology clinic patients. Consistently, Seetan et al. reported that the medical students' knowledge of skin cancer risk factors was relatively high [18]. The source of information about skin cancer was mostly books and magazines among medical students/GPs, which may be a reason for their higher knowledge because books and magazines are usually more reliable than Instagram and other social networks. Moreover, since skin cancer is a part of medical curriculum, it is reasonable that medical students or GPs would have better knowledge about skin cancer than the patients. On the other hand, the overall percentages show that more than half of the participants had good knowledge, but the majority lacked good attitude and practice toward skin cancer. What matters most in skin cancer prevention is in fact the performance and practice of sun-protective behaviors. In other words, higher knowledge and better attitude toward skin cancer do not necessarily lead to improved protection. This was reflected in the current study, where medical students/GPs had better knowledge and attitudes toward skin cancer but did not necessarily translate this into significantly better sun-protective practices compared to the patients.

A study conducted among young adults in the United States found that, similar to the current study, knowledge and attitudes were higher among healthcare providers (medical students) compared to the general population [19]. However, the American study reported better sun-protective practices among the younger population, in contrast to the current study's finding of better practices among the patient groups.

In the Middle Eastern context, a recent study in Saudi Arabia examined skin cancer KAP among university students and found generally low levels of knowledge and suboptimal sun-protective behaviors [20]. These findings align with the current study's results regarding the patient population's knowledge and practices. Further corroborating the current study's observations, these results are consistent with previous studies in the Middle East region, which have reported similar patterns of knowledge and practice among various populations [21–23].

Regarding healthcare providers, a study in Jordan found that physicians had relatively high levels of skin cancer knowledge, but their sun-protective practices were still suboptimal [18]. This is partially consistent with the current study's findings, where medical students/GPs had better knowledge but did not necessarily translate this into optimal sun-protective behaviors.

Turning to the Iranian context, literature review on perceptions and practices of the Iranian population regarding skin cancers reveals several key findings. The literature review on skin cancer perceptions and practices in Iran reveals a mixed picture—while skin cancer knowledge ranged from low to high across studies, there was a general pattern of low perceived susceptibility and severity of skin cancer. Overall, usage of sun protection methods like sunscreen, hats, and protective clothing was low [24].

Regarding healthcare providers in Iran, a study among Iranian physicians found that while they had good knowledge about skin cancer, their personal sun-protective practices were not optimal [25]. This mirrors the current study's observation of the discrepancy between medical students/GPs' knowledge/attitudes and their actual sun-protective behaviors.

Overall, Instagram was the most common skin cancer source of information in the current study; however, this was mostly attributable to the patients rather than medical students/GPs since among the latter, books and magazines were the most common information sources. The social and media pressure to appear tanned is significant. According to behavioral sciences, immediate advantages will have a greater impact on behaviors than prospective long-term losses [26]. Positive attitudes about tanning are more likely to affect behavior than just increasing skin cancer knowledge and understanding [27]. Similar to this, efforts that stir up strong emotions via personal experiences or unfavorable images perform better than knowledge-based ones [28]. Younger individuals are more likely to be motivated by appearance [29]. In harmony, the mean age of this study's participants shows that they were young. Therefore, creating and implementing behavior counseling targeted at appearance may be wise. A program like this could help individuals change their own opinions about tanning and sun protection [30].

The study findings revealed a positive correlation among knowledge, attitude, and practice regarding skin cancer among medical students/GPs, as anticipated. Interestingly, there was a negative correlation between knowledge and attitude among patients, with attitude being inversely correlated with their practice toward skin cancer. One possible explanation for the negative association between knowledge and attitude in this group is that as individuals become more informed about an illness, they may experience heightened fears, anxiety, and hypersensitivity toward any symptoms they experience, potentially leading to misinterpretation. As a result, this may lead to a negative impact on their demeanor, causing them to feel a lack of control over the situations and an inability to combat the disease.

There were no significant increases of odds with a history of skin cancer, and using sunscreens observed among the patient groups in the current study is an interesting finding that warrants further exploration. A recent study in Lebanon found a similar pattern [31]. This suggests that the relationship between KAP constructs may be more complex, particularly in certain populations, and requires deeper investigation.

Another finding of our study was that age had a positive correlation with attitude, practice, and overall KAP among patients, and with knowledge, practice, and overall KAP among medical students/GPs. Similarly, a study conducted in Turkey examined the performance of female oncology nurses on the Skin Cancer and Sun Knowledge (SCSK) scale. The study found that nurses who were above 40 years of age and had received education on skin cancer demonstrated better performance on the scale [32]. However, older patients of our study had significantly lower odds of good knowledge about skin cancer, which is in line with the findings of another study in Iran showing low skin cancer knowledge among the elderly [33]. Similarly, Mousavi et al.'s findings revealed that older adults had a low level of knowledge about skin cancer [34]. Of note, multiple studies have shown no significant relationship between age and KAP toward skin cancer [35, 36].

We also found that female patients and medical students/GPs had higher odds of good KAP compared to males, except for medical students/GPs' knowledge of skin cancer, which was not associated with gender. Consistently, a study on black and Hispanic individuals revealed that women exhibited greater knowledge and awareness of skin cancer, as well as a higher likelihood of engaging in sun-safe behaviors compared to men [37]. This can be attributed to women's tendency to pay more attention to their appearance, which is closely linked to the condition of their skin. Nonetheless, it is inaccurate to generalize that women prioritize their appearance more than men. Research suggests that women may exhibit greater body dissatisfaction and attach more significance to their physical appearance compared to men [38, 39]. However, it is crucial to acknowledge that these findings do not apply universally to all individuals of both genders. Age, culture, and interindividual differences influence individuals' emphasis on their appearance.

Among patients, although having a health-related occupation was not correlated with knowledge, it was significantly associated with attitude, practice, and overall KAP toward skin cancer. The lack of higher skin cancer knowledge among patients with health-related occupations can be attributed to the nature of their specific occupation. Not all health-related occupations involve contact with skin cancer patients or their care, and therefore may not have knowledge on this topic. On the other hand, GPs were less likely to have good practice and overall KAP toward skin cancer while having higher knowledge compared to medical students. This results in reduced adherence to sun protection practices during the transition from medical school to medical practice, highlighting the need for educational programs targeting GPs.

One interesting finding of the current study was that having a personal history of skin cancer was associated with reduced odds of good attitude, practice, and overall KAP in dermatology clinic patients, while the opposite was expected. This again points out to the gap of knowledge with attitude and more importantly practice of preventive measures against skin cancer. On the other hand, having a family history of skin cancer did not result into higher odds of good knowledge of skin cancer among the patients' group.

This study has some limitations. This study is not without limitations. The cross-sectional design limits the ability to establish causal relationships. Additionally, the study was conducted in a specific region of Iran, which may limit the generalizability of the findings to other geographic contexts. The study employed a self-report questionnaire. There is conflicting evidence regarding the accuracy of self-reported sun-protective practices. While some studies express doubts about its reliability, others have found that the recall of such practices is both reliable and valid [40, 41]. Furthermore, the absence of a universally accepted measure for assessing knowledge, attitude, and practice related to skin cancer in existing literature hinders the ability to compare findings [42]. Future research should employ longitudinal designs, incorporate objective measures of sun-protective behaviors, and expand the study to diverse populations within Iran and the broader Middle Eastern region.

In addition to the findings related to KAP, it is worth noting that recent advancements in image-based techniques, particularly the contributions of artificial intelligence (AI) and machine learning, have shown promising results in the early detection and diagnosis of melanoma. Studies have demonstrated the potential of deep learning algorithms for automated melanoma detection [43, 44], as well as the application of multiple instance learning approaches to improve the classification of dysplastic nevi [45, 46]. These technological advancements could potentially enhance skin cancer prevention and early intervention efforts by supporting clinicians and empowering patients to identify concerning lesions. Future research should explore how these innovative tools can be integrated into skin cancer education and prevention programs to complement the KAP-focused strategies.

## 4. Conclusions

More than three-fourths of the medical students/GPs had good knowledge, attitude, and overall KAP toward skin cancer, while good practice was only reported by one-third. On the other hand, although more than half of dermatology clinic patients had good knowledge of skin cancer, good attitude and practice were observed in less than one-third. These findings underscore the imperative need for prompt action to support the prevention of skin cancer. The current study's data lend credence to the idea that efforts to reduce the risk of skin cancer should place a greater emphasis on modifying attitudes and promoting sun-protective behaviors, particularly among the general populations.

Furthermore, the study's implications extend to healthcare professionals, as the results suggest the need to enhance skin cancer education and training, particularly in the areas of translating knowledge into practice. Implementing continuing medical education programs and developing practical skin cancer prevention guidelines could help bridge the gap between healthcare providers' knowledge and their personal sun-protective behaviors. Future research should explore the barriers and facilitators that influence the adoption of sun-safe practices among both medical professionals and the general public, to inform the design of more effective interventions.

Future public health initiatives should focus on strategies that positively shape attitudes and foster the adoption of sun-safe practices, in addition to strengthening knowledge. Tailoring these interventions to the specific characteristics and needs of each target group, whether patients or healthcare providers, will be crucial for driving meaningful improvements in skin cancer prevention and early detection. Moreover, the study underscores the potential role of emerging technologies, such as AI-powered image analysis for automated melanoma detection, which can play in complementing KAP-focused efforts. Integrating these advanced tools into skin cancer education and prevention programs may empower both clinicians and the general public to identify concerning lesions early, ultimately contributing to better health outcomes. By addressing the gaps in attitudes and practices, while harnessing the power of technological advancements, public health stakeholders can develop comprehensive strategies to effectively translate skin cancer knowledge into sustained, evidence-based preventive actions. This multifaceted approach holds promise for reducing the growing burden of skin cancer and improving the overall well-being of the population.

In conclusion, this study provides important insights into the current state of skin cancer-related KAP among dermatology patients and medical professionals in Iran. By comparing the findings with recent literature, both globally, within the Middle Eastern context, and specifically in Iran, the authors have highlighted the similarities and differences in skin cancer KAP, as well as the need for targeted interventions to improve prevention practices, particularly among the patient populations. The insights from this study can inform the development of effective public health strategies and educational programs to enhance skin cancer awareness and promote comprehensive sun-protective behaviors in this region, ultimately contributing to the prevention of this increasingly prevalent malignancy. Longitudinal studies examining the long-term impacts of such interventions would be a valuable next step to further strengthen the evidence base for skin cancer prevention in this context.

## Figures and Tables

**Table 1:** General characteristics of the participants.

Variables	Patients (*n* = 1953)	Medical students/GPs (*n* = 290)	Total (*n* = 2243)
Age (years), mean (SD)	28.79 (8.83)	25.51 (4.87)	28.37 (8.49)
Sex, *N* (%)			
Male	948 (48.5)	109 (37.6)	1057 (47.1)
Female	1005 (51.5)	181 (62.4)	1186 (52.9)
Education, *N* (%)			
Under diploma	206 (10.5)	—	206 (9.2)
Diploma—early semesters (0–5)	405 (20.7)	13 (4.48)	418 (21.4)
Bachelor's—middle semesters (6–10)	652 (33.4)	137 (47.24)	789 (29.6)
Master's—late semesters (10-11)	552 (28.3)	77 (26.55)	629 (25.1)
PhD—general practitioners	138 (7.1)	63 (21.72)	201 (14.7)
Semester^*∗*^, mean (SD)	—	10.21 (2.91)	10.21 (2.91)
Medical field-related occupations, *N* (%)			
Yes	52 (2.7)	290 (100)	342 (15.24)
No	1901 (97.3)	0 (0)	1901 (84.75)
Do patients ask you questions about health issues^†^, *N* (%)			
Yes	36 (69.2)	230 (87.5)	266 (84.4)
No	16 (30.8)	33 (12.5)	49 (15.6)
Personal history of skin cancer, *N* (%)			
Yes	63 (3.2)	7 (2.4)	70 (3.1)
No	1890 (96.8)	283 (97.6)	2173 (96.9)
Family history of skin cancer, *N* (%)			
Yes	76 (3.9)	7 (2.4)	83 (3.7)
No	1877 (96.1)	283 (97.6)	2160 (96.3)
Skin cancer source of information, *N* (%)			
Family and friends	1445 (74.0)	21 (7.2)	1466 (65.4)
Radio and TV	1322 (67.7)	37 (12.8)	1359 (60.6)
Instagram	1585 (81.2)	62 (21.4)	1647 (73.4)
Other social networks	1258 (64.4)	55 (18.9)	1313 (58.5)
Books and magazines	1270 (65.0)	205 (70.7)	1475 (65.8)
Medical staff	1084 (55.5)	166 (57.2)	1250 (55.7)
Fitzpatrick skin type, *N* (%)			
I	535 (27.4)	21 (7.2)	556 (24.8)
II	504 (25.8)	119 (41.0)	623 (27.8)
III	552 (28.3)	121 (41.7)	673 (30.0)
IV	222 (11.4)	28 (9.7)	250 (11.1)
V	140 (7.2)	1 (0.3)	141 (6.3)

^*∗*^For medical students (*n* = 233). ^†^Of those having medical field-related occupations (*n* = 315).

**Table 2:** Comparison of the mean scores of knowledge, attitude, and practice of patients and medical students/GPs toward skin cancer.

Variables	Patients (*n* = 1953)	Medical students/GPs (*n* = 290)	Total (*n* = 2243)	*P* value^*∗*^
Knowledge	16.35 (2.89)	19.73 (4.19)	16.79 (8.29)	<0.001
Attitude	4.65 (2.47)	11.44 (2.47)	5.53 (3.36)	<0.001
Practice	13.49 (3.98)	12.16 (5.08)	13.31 (4.16)	<0.001
Total	34.49 (6.13)	43.33 (8.90)	35.63 (7.19)	<0.001

Variables are expressed as mean (SD). ^*∗*^Analysis using the independent *t*-test.

**Table 3:** The correlation of knowledge, attitude, and practice toward skin cancer.

	First variable	Second variable	Correlation coefficient	*P* value^*∗*^
Total	Knowledge	Attitude	0.103	<0.001
		Practice	0.245	<0.001
	Attitude	Practice	−0.072	0.001

Patients	Knowledge	Attitude	−0.142	<0.001
		Practice	0.298	<0.001
	Attitude	Practice	−0.062	<0.001

Medical students (GPs)	Knowledge	Attitude	0.329	<0.001
		Practice	0.268	<0.001
	Attitude	Practice	0.319	<0.001

^*∗*^Analysis using the Spearman's Rho.

**Table 4:** Logistic regression analysis to determine the association of knowledge, attitude, practice, and overall KAP toward skin cancer with participants' general characteristics.

Variables	Knowledge		Attitude		Practice		KAP	
	aOR (95% CI)	*P* value	aOR (95% CI)	*P* value	aOR (95% CI)	*P* value	aOR (95% CI)	*P* value
*Patients*								
Age	0.973 (0.959; 0.986)	<0.001	1.022 (1.001; 1.043)	0.045	1.082 (1.066; 1.098)	<0.001	1.127 (1.109; 1.146)	<0.001
Sex								
Male	1.000	—	1.000	—	1.000	—	1.000	—
Female	1.773 (1.457; 2.159)	<0.001	2.432 (1.688; 3.503)	<0.001	2.908 (2.314; 3.655)	<0.001	1.730 (1.347; 2.222)	<0.001
Education								
Diploma or lower	1.000	—	1.000	—	1.000	—	1.000	—
University	3.802 (2.955; 4.891)	<0.001	1.657 (1.059; 2.594)	0.027	1.715 (1.287; 2.285)	<0.001	0.971 (0.703; 1.342)	0.859
Health-related occupation	—	—	29.731 (15.124; 58.447)	<0.001	1.854 (0.991; 3.469)	0.053	6.711 (3.498; 12.874)	<0.001
Personal history of skin cancer	79.530 (10.771; 587.232)	<0.001	0.114 (0.014; 0.948)	0.045	0.036 (0.008; 0.165)	<0.001	0.025 (0.003; 0.194)	<0.001
Family history of skin cancer	0.147 (0.077; 0.281)	<0.001	—	—	20.747 (10.135; 42.473)	<0.001	2.281 (1.318; 3.947)	0.003

*Medical students (GPs)*								
Age	1.170 (1.015; 1.348)	0.030	—	—	1.115 (1.025; 1.212)	0.011	1.168 (1.020; 1.338)	0.024
Sex								
Male	1.000	—	1.000	—	1.000	—	1.000	—
Female	—	—	3.780 (1.633; 8.752)	0.002	9.324 (4.456; 19.508)	<0.001	3.984 (2.208)	<0.001
Education								
Medical student	1.000	—	1.000	—	1.000	—	1.000	—
GP	1.207 (0.359; 4.063)	0.761	—	—	0.927 (0.391; 2.200)	0.863	0.647 (0.213; 1.965)	0.443
Personal history of skin cancer	—	—	—	—	3.988 (0.546; 29.139)	0.173	—	—
Family history of skin cancer	—	—	—	—	17.113 (1.699; 172.382)	0.016	—	—

aOR, adjusted odds ratio; CI, confidence interval; GP, general practitioner; KAP, knowledge, attitude, and practice.

## Data Availability

The datasets used and/or analyzed during the current study are available from the corresponding author upon reasonable request.

## Supplementary Materials

Supplementary Materials Supplementary Table 1: Knowledge about skin cancer. Supplementary Table 2: Attitude toward skin cancer. Supplementary Table 3: Practice toward skin cancer.
