# Supplementary material for: Knowledge, Attitude, and Practice toward Skin Cancer among Patients of Dermatology Clinics and Medical Students/General Practitioners
Source: J Skin Cancer. 2024 May 20;2024:9081896. doi: 10.1155/2024/9081896 (PMC11129908; doi:10.1155/2024/9081896)
Supplement: Supplementary Materials — Supplementary Table 1: Knowledge about skin cancer. Supplementary Table 2: Attitude toward skin cancer. Supplementary Table 3: Practice toward skin cancer. [file 9081896.f1.docx]

Supplementary Table 1: Knowledge about skin cancer

| Items | Outpatients (n=1953) | Medical students/GPs (n=290) | Total (n=2243) |
| --- | --- | --- | --- |
| Sun exposure can cause skin cancer. |  |  |  |
| Correct | 1225 (62.7) | 244 (84.1) | 1469 (65.5) |
| Incorrect | 452 (23.1) | 32 (11.0) | 484 (21.6) |
| Don’t know | 276 (14.1) | 14 (4.8) | 290 (12.9) |
| Life-long risk of skin cancer |  |  |  |
| 0% | 16 (0.8) | 3 (1.0) | 19 (0.8) |
| 0-5% | 252 (12.9) | 111 (38.3) | 363 (16.2) |
| 5-10% | 385 (19.7) | 64 (22.1) | 449 (20.0) |
| 10-15% | 567 (29.0) | 57 (19.7) | 624 (27.8) |
| 15-30% | 573 (29.3) | 35 (19.7) | 608 (27.1) |
| 30-60% | 156 (8.0) | 17 (5.9) | 173 (7.7) |
| 60-90% | 4 (0.2) | 3 (1.0) | 7 (0.3) |
| The greatest carcinogenic effect of sunlight |  |  |  |
| Direct sunlight to the skin | 959 (49.1) | 188 (64.8) | 1147 (51.1) |
| Sunlight shining through the glass | 657 (33.6) | 20 (6.9) | 677 (30.2) |
| No difference | 275 (14.1) | 63 (21.7) | 338 (15.1) |
| Don’t know | 62 (3.2) | 19 (6.6) | 81 (3.6) |
| The group in which skin cancer is more common |  |  |  |
| Individuals with light skin | 618 (31.6) | 226 (77.9) | 844 (37.6) |
| Individuals with dark skin | 639 (32.7) | 21 (7.2) | 660 (29.4) |
| Not associated with skin color | 530 (27.1) | 21 (7.2) | 551 (24.6) |
| Don’t know | 166 (8.5) | 22 (7.6) | 188 (8.4) |
| Changes in the sun’s carcinogenic rays in the cloudy days of winter compared to the sunny days |  |  |  |
| Increases | 398 (20.4) | 99 (34.1) | 497 (22.2) |
| Decreases | 702 (35.9) | 77 (26.6) | 779 (34.7) |
| No change | 298 (15.3) | 114 (39.3) | 412 (18.4) |
| Doesn’t exist | 555 (28.4) | 0 (0.0) | 555 (24.7) |
| Daytime with the highest UVR |  |  |  |
| 8-11 AM | 168 (8.6) | 20 (6.9) | 188 (8.4) |
| 10 AM - 3 PM | 1219 (62.4) | 231 (79.7) | 1450 (64.6) |
| 3-6 PM | 437 (22.4) | 23 (7.9) | 460 (20.5) |
| No difference | 129 (6.6) | 16 (5.5) | 145 (6.5) |
| Changes to the carcinogenic effects of sunlight in the shadow or indoors |  |  |  |
| Disappears completely | 611 (31.3) | 23 (7.9) | 634 (28.3) |
| Increases | 502 (25.7) | 3 (1.0) | 505 (22.5) |
| Decreases | 649 (33.2) | 225 (77.6) | 874 (39.0) |
| No change | 191 (9.8) | 39 (13.4) | 230 (10.3) |
| Factors that increase the risk of skin cancer |  |  |  |
| Little but constant exposure to sunlight during lifetime | 1591 (81.5) | 128 (44.1) | 1719 (76.6) |
| Darker skin | 792 (40.6) | 22 (7.6) | 814 (36.3) |
| Having more/multiple moles | 1559 (79.8) | 132 (45.5) | 1691 (75.4) |
| Children playing in the sunlight | 827 (42.3) | 81 (27.9) | 908 (40.5) |
| Having freckles | 1083 (55.5) | 45 (15.5) | 1128 (50.3) |
| Skin cancer in first-degree relatives | 736 (37.7) | 253 (87.2) | 989 (44.1) |
| Jobs with long working hours in the sun | 281 (14.4) | 256 (88.3) | 537 (23.9) |
| Tanned and sunburned skin | 416 (14.4) | 139 (47.9) | 555 (24.7) |
| Viruses that can cause skin cancer |  |  |  |
| Human immunodeficiency virus | 125 (6.4) | 72 (24.8) | 197 (8.8) |
| Hepatitis B | 327 (16.7) | 21 (7.2) | 348 (15.5) |
| The virus that causes warts | 382 (19.6) | 164 (56.6) | 546 (24.3) |
| Don’t know | 1257 (64.4) | 85 (29.3) | 1342 (59.8) |
| Viruses have no effect on the occurrence of skin cancer | 390 (20.0) | 15 (5.2) | 405 (18.1) |
| What changes in moles may indicate cancer |  |  |  |
| Enlargement | 1464 (75.0) | 232 (80.0) | 1696 (75.6) |
| Discoloration | 1260 (64.5) | 255 (87.9) | 1515 (67.5) |
| Bleeding | 1320 (67.6) | 166 (57.2) | 1486 (66.3) |
| Don’t know | 214 (11.0) | 15 (5.2) | 229 (10.2) |
| Effective measures to prevent skin cancer |  |  |  |
| Avoiding sunlight | 1058 (54.3) | 154 (53.5) | 1212 (54.2) |
| Wearing long-sleeved clothes | 553 (28.4) | 209 (72.6) | 762 (34.0) |
| Using hats and masks | 1676 (85.9) | 224 (77.8) | 1900 (84.9) |
| Using sunscreen | 1561 (80.1) | 279 (96.9) | 1840 (82.2) |
| None | 98 (5.0) | 2 (0.7) | 100 (4.5) |
| Effectiveness of sunscreens in skin cancer prevention |  |  |  |
| 100% prevent skin cancer | 758 (38.8) | 12 (4.1) | 770 (34.3) |
| Prevent skin cancer to some extent | 877 (44.9) | 272 (93.8) | 1149 (51.2) |
| No effect on skin cancer | 318 (16.3) | 6 (2.1) | 324 (14.4) |
| Maximum duration of sunscreen effects |  |  |  |
| 2-3 hours | 1174 (60.1) | 231 (79.7) | 1405 (62.6) |
| Half a day | 394 (20.2) | 39 (13.4) | 433 (19.3) |
| All day | 284 (14.5) | 10 (3.4) | 294 (13.1) |
| Don’t know | 101 (5.2) | 10 (3.4) | 111 (4.9) |
| SPF for sunscreens |  |  |  |
| At least 30 | 1464 (75.0) | 169 (58.3) | 1633 (72.8) |
| At least 60 in areas like our country | 1532 (78.4) | 92 (31.7) | 1624 (72.4) |
| The higher SPF, the stronger sun protection | 496 (25.4) | 79 (27.2) | 575 (25.6) |
| It only has a commercial aspect | 165 (8.4) | 12 (4.1) | 177 (7.9) |
| The higher SPF, the more harmful | 3 (0.2) | 4 (1.4) | 7 (0.3) |
| Don’t know | 63 (3.2) | 30 (10.3) | 93 (4.1) |
| Sunscreens … |  |  |  |
| Reduce sunburn | 1742 (89.2) | 258 (89.0) | 2000 (89.2) |
| Reduce skin cancer occurrence | 1080 (55.3) | 246 (84.8) | 1326 (59.1) |
| Reduce premature skin aging and wrinkles | 1727 (88.4) | 213 (73.4) | 1940 (86.5) |
| Reduce the occurrence of new spots | 457 (23.4) | 202 (69.7) | 659 (29.4) |
| Have no effect | 131 (6.7) | 2 (0.7) | 133 (5.9) |
| Contain harmful chemicals | 103 (5.3) | 25 (8.6) | 128 (5.7) |

All values are expressed as N (%).

Supplementary Table 2: Attitude towards skin cancer

| Items | Outpatients (n=1953) | Medical students/GPs (n=290) | Total (n=2243) |
| --- | --- | --- | --- |
| Tanned and sunburned skin indicates health and well-being. |  |  |  |
| Agree | 794 (40.7) | 13 (4.5) | 807 (36.0) |
| Neutral | 898 (46.0) | 58 (20.0) | 956 (42.6) |
| Disagree | 261 (13.4) | 219 (75.5) | 480 (21.4) |
| Tanned and sunburned skin improves a person’s appearance. |  |  |  |
| Agree | 541 (27.7) | 46 (15.9) | 587 (26.2) |
| Neutral | 1019 (52.2) | 73 (25.2) | 1092 (48.7) |
| Disagree | 393 (20.1) | 171 (59.0) | 564 (25.1) |
| Tanned and sunburned skin is worth increasing the risk of skin cancer. |  |  |  |
| Agree | 606 (31.0) | 104 (35.9) | 710 (31.7) |
| Neutral | 912 (46.7) | 26 (9.0) | 938 (41.8) |
| Disagree | 435 (22.3) | 160 (55.2) | 595 (26.5) |
| Skin cancer is usually a fatal disease. |  |  |  |
| Agree | 858 (43.9) | 101 (34.8) | 959 (42.8) |
| Neutral | 840 (43.0) | 62 (21.4) | 902 (40.2) |
| Disagree | 255 (13.1) | 127 (43.8) | 382 (17.0) |
| Skin cancer is more common than other cancers. |  |  |  |
| Agree | 707 (36.2) | 106 (36.6) | 813 (36.2) |
| Neutral | 1027 (52.6) | 71 (24.5) | 1098 (49.0) |
| Disagree | 219 (11.2) | 113 (39.0) | 332 (14.8) |
| I will never get skin cancer and I don’t consider myself at risk. |  |  |  |
| Agree | 979 (50.1) | 41 (14.1) | 1020 (45.5) |
| Neutral | 711 (36.4) | 92 (31.7) | 803 (35.8) |
| Disagree | 263 (13.5) | 157 (54.1) | 420 (18.7) |
| Skin cancer is preventable. |  |  |  |
| Agree | 1561 (79.9) | 259 (89.3) | 1820 (81.1) |
| Neutral | 287 (14.7) | 28 (9.7) | 315 (14.0) |
| Disagree | 105 (5.4) | 3 (1.0) | 108 (4.8) |
| Sun protection 100% prevents skin cancer. |  |  |  |
| Agree | 1034 (52.9) | 21 (7.2) | 1055 (47.0) |
| Neutral | 274 (14.0) | 40 (13.8) | 314 (14.0) |
| Disagree | 645 (33.0) | 229 (79.0) | 874 (39.0) |
| Only those who have a family history of skin cancer get skin cancer. |  |  |  |
| Agree | 690 (35.3) | 2 (0.7) | 692 (30.9) |
| Neutral | 757 (38.8) | 10 (3.4) | 767 (34.2) |
| Disagree | 506 (25.9) | 278 (95.9) | 784 (35.0) |
| Skin cancer usually occurs in old age. |  |  |  |
| Agree | 500 (25.6) | 132 (45.5) | 632 (28.2) |
| Neutral | 463 (23.7) | 55 (19.0) | 518 (23.1) |
| Disagree | 990 (50.7) | 103 (35.5) | 1093 (48.7) |
| Exposure to sunlight to tan the skin can contribute to the occurrence of skin cancer. |  |  |  |
| Agree | 869 (44.5) | 257 (88.6) | 1126 (50.2) |
| Neutral | 219 (11.2) | 22 (7.6) | 241 (10.7) |
| Disagree | 865 (44.3) | 11 (3.8) | 876 (39.1) |
| Using sunscreen to prevent skin cancer is time-consuming. |  |  |  |
| Agree | 922 (47.2) | 30 (10.3) | 952 (42.4) |
| Neutral | 368 (18.8) | 20 (6.9) | 388 (17.3) |
| Disagree | 663 (33.9) | 240 (82.8) | 903 (40.3) |
| I don’t use protective equipment (hats, gloves, sunglasses, etc.) because it attracts the attention of others. |  |  |  |
| Agree | 614 (31.4) | 51 (17.6) | 665 (29.6) |
| Neutral | 979 (50.1) | 34 (11.7) | 1013 (45.2) |
| Disagree | 360 (18.4) | 205 (70.7) | 565 (25.2) |
| I feel that the use of protective equipment doesn’t have much effect on skin cancer prevention. |  |  |  |
| Agree | 1300 (66.6) | 13 (4.5) | 1313 (58.5) |
| Neutral | 423 (21.7) | 20 (6.9) | 443 (19.8) |
| Disagree | 230 (11.8) | 257 (88.6) | 487 (21.7) |
| I feel that the use of sunscreen doesn’t have much effect on skin cancer prevention. |  |  |  |
| Agree | 1262 (64.6) | 12 (4.1) | 1274 (56.8) |
| Neutral | 311 (15.9) | 11 (3.8) | 322 (14.4) |
| Disagree | 380 (19.5) | 267 (92.1) | 647 (28.8) |
| I believe that I should protect myself from skin cancer by taking protective measures against the sun. |  |  |  |
| Agree | 1062 (54.4) | 255 (87.9) | 1317 (58.7) |
| Neutral | 657 (33.6) | 25 (8.6) | 682 (30.4) |
| Disagree | 234 (12.0) | 10 (3.4) | 244 (10.9) |

All values are expressed as N (%).

Supplementary Table 3: Practice towards skin cancer

| Items | Outpatients (n=1953) | Medical students/GPs (n=290) | Total (n=2243) |
| --- | --- | --- | --- |
| Ever tried to personally get information about skin cancer from different sources |  |  |  |
| Yes | 1313 (67.2) | 154 (53.1) | 1467 (65.4) |
| No | 640 (32.8) | 136 (46.9) | 776 (34.6) |
| If they sunbathe and tan |  |  |  |
| Yes | 1356 (69.4) | 21 (7.2) | 1377 (61.4) |
| No | 597 (30.6) | 269 (92.8) | 866 (38.6) |
| The effort they make to do things during the time of the day when there is less sun exposure (except sports) |  |  |  |
| Never | 98 (5.0) | 32 (11.0) | 130 (5.8) |
| <25% (1 point) | 575 (29.4) | 94 (32.4) | 669 (29.8) |
| 25-50% (2 points) | 1079 (55.2) | 64 (22.1) | 1143 (51.0) |
| 50-75% (3 points) | 64 (3.3) | 58 (20.0) | 122 (5.4) |
| >75% (4 points) | 137 (7.0) | 42 (14.5) | 179 (8.0) |
| The effort they make to do sports activities during the time of the day when there is less sun exposure |  |  |  |
| Never | 290 (14.8) | 27 (9.3) | 317 (14.1) |
| <25% (1 point) | 263 (13.5) | 57 (19.7) | 320 (14.3) |
| 25-50% (2 points) | 61 (3.1) | 36 (12.4) | 97 (4.3) |
| 50-75% (3 points) | 1224 (62.7) | 62 (21.4) | 1286 (57.3) |
| >75% (4 points) | 115 (5.9) | 108 (37.2) | 223 (9.9) |
| The protective equipment they use to protect themselves from the sun |  |  |  |
| Sunscreen | 1783 (91.3) | 192 (66.2) | 1975 (88.1) |
| Sunglasses | 1035 (53.0) | 200 (69.0) | 1235 (55.1) |
| Gloves | 1447 (74.1) | 24 (8.3) | 1471 (65.6) |
| Cap/hat | 1441 (73.8) | 73 (25.2) | 1514 (67.5) |
| None | 26 (1.3) | 34 (11.7) | 60 (2.7) |
| When they use sunscreen |  |  |  |
| Every day they leave home (3 points) | 820 (42.0) | 140 (48.3) | 960 (42.8) |
| Only on sunny days (2 points) | 807 (41.3) | 38 (13.1) | 845 (37.7) |
| Only on summer days (2 points) | 624 (32.0) | 21 (7.2) | 645 (28.8) |
| Don’t use sunscreen | 1278 (65.4) | 78 (26.9) | 1356 (60.5) |
| Before doing sports and going outdoors (1 point) | 1025 (52.5) | 66 (22.8) | 1091 (48.6) |
| If they use sunscreen |  |  |  |
| No | 1079 (55.2) | 88 (30.3) | 1167 (52.0) |
| Yes, to prevent sunburn | 485 (24.8) | 176 (60.7) | 661 (29.5) |
| Yes, to prevent skin cancer | 527 (27.0) | 127 (43.8) | 654 (29.2) |
| Yes, to prevent premature skin aging and wrinkles | 632 (32.4) | 129 (44.5) | 761 (33.9) |
| Yes, to prevent the occurrence of new spots | 496 (25.4) | 109 (37.6) | 605 (27.0) |
| How often they use sunscreen |  |  |  |
| Every 2-3 hours | 759 (38.9) | 46 (15.9) | 805 (35.9) |
| Every 6 hours | 432 (22.1) | 35 (12.1) | 467 (20.8) |
| Every 12 hours | 270 (13.8) | 9 (3.1) | 279 (12.4) |
| Only once a day | 369 (18.9) | 115 (39.7) | 484 (21.6) |
| Don’t use sunscreen | 123 (6.3) | 85 (29.3) | 208 (9.3) |
| If they renew sunscreen after washing their hands and face |  |  |  |
| Yes | 467 (23.9) | 178 (61.4) | 645 (28.8) |
| No | 1486 (76.1) | 112 (38.6) | 1598 (71.2) |

All values are expressed as N (%).
